# The prognostic value of programmed cell death protein ligand 1 in patients with non-muscle-invasive bladder cancer treated with bacille Calmette–Guérin immunotherapy: Current status

**DOI:** 10.1080/2090598X.2020.1791562

**Published:** 2020-07-16

**Authors:** Łukasz Nowak, Wojciech Krajewski, Adrian Poterek, Anna Śliwa, Romuald Zdrojowy

**Affiliations:** Department of Urology and Urological Oncology, Wroclaw Medical University, Wroclaw, Poland

**Keywords:** Bladder cancer, NMIBC, PD-L1, BCG, immunotherapy

## Abstract

**Objective:**

To summarise the current evidence of the significance and prognostic value of programmed cell death protein ligand 1 (PD-L1) expression in patients with non-muscle-invasive bladder cancer (NMIBC) treated with bacille Calmette–Guérin (BCG) immunotherapy.

**Methods:**

A search was conducted in May 2020 of three electronic databases; MEDLINE, Scopus, and EMBASE. In this review we included results from original studies investigating the relationship between the PD-L1 expression and BCG response in patients with NMIBC.

**Results:**

Only five relevant articles were identified in the literature to date. Some studies showed an association between increased PD-L1 expression and BCG unresponsiveness; however, other authors provided contradictory results and suggested that PD-L1 evaluation could not be used for reliable prediction of BCG response.

**Conclusions:**

The value of PD-L1 evaluation in predicting BCG response is debatable. Current evidence, based only on retrospective analyses, is inconsistent. Comparability of the results is diminished by the methodological limitations of immunohistochemistry assessment. Further multicentre, randomised trials are needed to make definitive conclusions.

**Abbreviations:**

ICs: immune cells; IHC: immunohistochemical staining; (N)MIBC: (non-) muscle-invasive bladder cancer; PD-L1: programmed cell death protein ligand 1; PD-1: programmed cell death protein 1; RC: radical cystectomy; TCs: tumour cells.

## Introduction

In the management of high-risk non-muscle-invasive bladder cancer (NMIBC), adjuvant intravesical BCG immunotherapy has been considered the ‘gold standard’ for several decades [1]. Nevertheless, the clinical utility and oncological outcomes of BCG treatment are impaired by the fact that eventually 30–40% of patients experience tumour recurrence and 15–20% of patients progress to muscle-invasive disease (muscle-invasive bladder cancer [MIBC]) [2]. Radical cystectomy (RC) remains the preferred treatment in patients who fail to respond to BCG, with limited data for bladder-sparing approaches such as tri-modality treatment [2]. However, performing RC is potentially related to severe complications and considerable mortality. Thus, it can be considered as overtreatment in some individuals [1,2]. Early prediction of patients who might not benefit from BCG and demand immediate radical treatment is crucial, but precise identification of such patients has been challenging to date.

The ability of the programmed cell death protein ligand 1 (PD-L1) to predict response to therapy has been investigated in various solid tumour types [3]. PD-L1 is a cell surface co-stimulatory glycoprotein, which can be expressed on tumour cells (TCs) and immune cells (ICs) infiltrating the tumour microenvironment. In the setting of cancer, the excessive activation of PD-L1 pathway can lead to T-cell dysfunction and exhaustion, resulting in decreased cytotoxic activity and ineffective targeting of TCs [3]. In clinical practice, PD-L1 expression is measured predominantly by immunohistochemical staining (IHC). PD-L1 levels can be evaluated separately in TCs and tumour-infiltrating ICs, or simultaneously in both cell populations [3].

In bladder cancer, the prognostic value of PD-L1 has been investigated primarily in relation to MIBC. It was shown that high PD-L1 expression could be associated with worse clinical and oncological outcomes [4]. In patients with NMIBC, PD-L1 has been suggested as a potential predictor of BCG response. Therefore, in the present review we wanted to briefly summarise the current evidence of the prognostic value of PD-L1 in patients with NMIBC treated with BCG.

## Methods

A search was conducted in May 2020 of the three online databases; the Medical Literature Analysis and Retrieval System Online (MEDLINE), Scopus and the Excerpta Medica dataBASE (EMBASE), according to Preferred Reporting Items for Systematic Reviews and Meta-Analysis (PRISMA) statement [5]. The Medical Subject Heading (MeSH) terms and/or keywords and/or free words were: ‘bladder cancer’, ‘non-muscle invasive’, ‘NMIBC’, ‘PD-L1ʹ, ‘PDL1ʹ, ‘BCG’, and ‘immunotherapy’. Boolean operators (NOT, AND, OR) were used in succession to narrow and broaden the search. Auto-alerts in MEDLINE were also run, as well as reference lists of original articles and review articles for further eligible data. The search included articles without language limitations and evidence was limited to human data.

**Figure 1.**
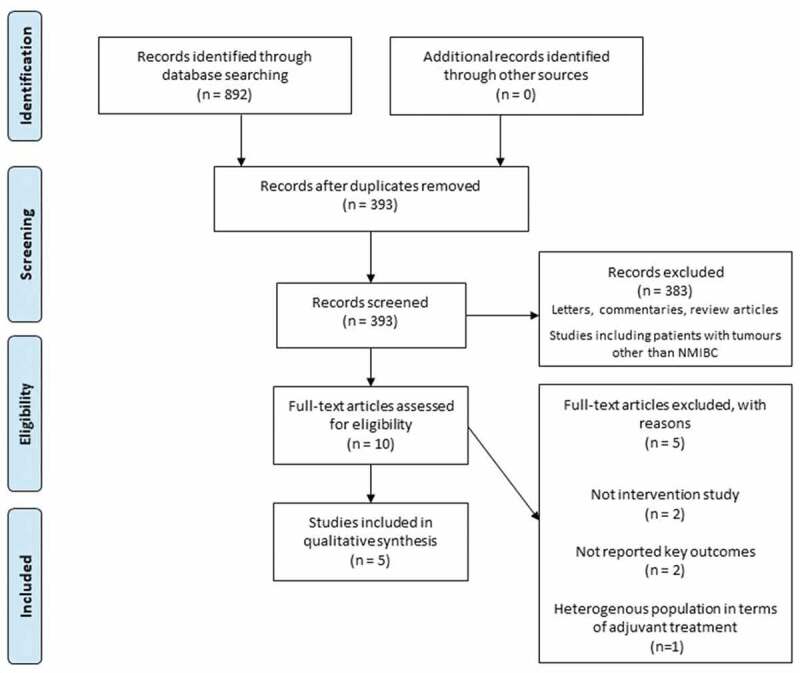
Flow diagram of study selection

In total, 892 publications were initially identified through database searching. The flow diagram of the study selection process with subsequent exclusions (with reasons) is presented in Figure 1. In the present review, we included results from original comparative studies investigating the relationship between the PD-L1 expression and BCG response in patients with NMIBC.

## Results

Only relevant five articles were identified in the literature to date. The characteristic and primary findings of available studies are summarised in Table 1 [6–10]. In an analysis of 22 patients with high-risk NMIBC, Martínez et al. [6] found that pre-treatment PD-L1 expression did not differ significantly between patients classified as BCG ‘responders’ and ‘non-responders’, defined as patients without a recurrence or progression for ≥30 months after BCG treatment initiation. Positive PD-L1 expression was observed in nine of the 12 BCG non-responders and seven of the 10 responders. Delcourt et al. [7] investigated the association between early recurrence (the occurrence of refractory tumour as defined by International Bladder Cancer Group [IBCG] and American Society of Clinical Oncology Genitourinary Group [ASCO GU] definitions) and the PD-L1 expression in a large cohort of 186 patients with high-risk NMIBC. The rate of early recurrence among patients with positive and negative pre-treatment PD-L1 expressions on TCs was 20% (seven of 35) and 20.5% (31/151), respectively (not significant). Similarly, no significant association was found when tumour infiltrating ICs were analysed. Additionally, the authors showed that the PD-L1 expression was significantly increased after BCG installations compared to the pre-treatment level. In another paper, including patients with high-risk NMIBC, Kates et al. [8] reported a significantly increased PD-L1 expression among pre-treatment samples collected from 32 BCG ‘non-responders’ (BCG unresponsive, relapsing progressors) compared to 31 ‘responders’. The positive PD-L1 expression was observed in 25–28% and 0–4%, respectively. PD-L1 expression, evaluated after BCG treatment, did not change significantly. Aydin et al. [9] analysed the pre-treatment PD-L1 expression in 117 patients with high-grade NMIBC. The authors found a significant association between positive PD-L1 expression on tumour infiltrating ICs and refractory recurrence (defined according to criteria specified in European Association of Urology [EAU] guidelines). However, no correlation was found regarding relapsing recurrence or progression. Also, PD-L1 expression was not a significant predictor of recurrence-free survival (RFS) or progression-free survival (PFS) in multivariate Cox regression analysis. Post-treatment PD-L1 expression levels on ICs were significantly decreased in patients who had refractory recurrence. In the most recent study, Pierconti et al. [10] compared PD-L1 expression between BCG unresponsive patients and those who responded to BCG therapy. Only patients with primary carcinoma *in situ* (CIS) were included in this study. The authors showed that the PD-L1 expression on both TCs and tumour infiltrating ICs was significantly higher in BCG unresponsive patients.

**Table 1.** Characteristic and primary findings of the presented studies

Reference	Design	No. of patients	Tumours (NMIBC)	BCG course	Analysed cell population	Assay*	PD-L1 positivity threshold, %	Pre-treatment positive PD-L1 expression, %	Findings
Martínez et al., [6]	R	22	T1 high-grade	Induction and maintenance	TCs, tumour infiltrating ICs	SP142	≥1	TCs: 9 ICs: 77	No significant difference in the expression pattern of PD-L1 was identified between the BCG responders and BCG non-responders^a^.
Delcourt et al., [7]	R	186	High-risk	Induction	TCs, tumour infiltrating ICs	E1L3 N	≥1	TCs: 18.1 ICs: 58.1	Early recurrence^b^ was not significantly more frequent in the PD-L1-positive patients vs the PD-L1-negative patients. PD-L1 expression was significantly increased on ICs after BCG therapy.
Kates et al., [8]	R	63	High-risk	Induction	TCs, tumour infiltrating ICs	SP142, 22C3	≥5	CPS: SP-142: 25 22C3: 28	PD-L1 was significantly increased in the pre-treatment samples collected from BCG non-responders^b^ vs BCG responders. BCG treatment did not increase PD-L1 expression.
Aydin et al. [9]	R	117	High grade	At least induction	TCs, tumour infiltrating ICs	SP142	≥1	TCs: 8.5 ICs: 77	Positive pre-treatment PD-L1 expression (on ICs) was significantly associated with refractory tumour recurrence^c^ but not with relapsing recurrence^c^ and progression^c^. RFS and PFS did not correlate with positive PD-L1 expression. Post-treatment PD-L1 expression levels on ICs were significantly decreased in patients who had refractory recurrence.
Pierconti et al., [10]	R	60	Primary CIS	At least induction	TCs, tumour infiltrating ICs	SP263, 22C3, SP142	Low (0–5) High (>5)	TCs: 16.7 ICs: 38.3	PD-L1 expression on TCs and on ICs was higher in BCG-unresponsive^c^ patients vs BCG responders, but only the PD-L1 22C3 expression in TCs was associated with recurrence of disease.

CPS, combined positivity score; R, retrospective.

* In all studies PD-L1 expression was evaluated by IHC.

^a^Defined as patients without a recurrence or progression based on follow-up cystoscopy and urinary cytology ≥30 months after BCG treatment initiation.

^b^Defined by IBCG and GU ASCO definitions

^c^Defined according to criteria specified in EAU Guidelines,

## Discussion

The value of PD-L1 as a predictive biomarker for BCG response is questionable. Although some studies have shown a positive correlation between increased PD-L1 expression and BCG unresponsiveness, results could be biased because of the small patient cohorts [8,10]. Studies with a large number of participants provided contradictory conclusions and suggested that evaluating PD-L1 expression could not be used for the prediction of BCG response [7,9]. The high level of inconsistency among the available studies may result from fact that PD-L1 expression was evaluated by IHC and multiple assays with different antibody clones were used. Moreover, the intra-tumoral heterogeneity of PD-L1 expression and the possibility of continuous changes in PD-L1 expression due to the animated nature of the tumour microenvironment could also distort the results and limit the interchangeability and comparability of particular studies. To overcome limitations of IHC method, the measurement of PD-L1 mRNA level by quantitative reverse transcriptase-polymerase chain reaction was suggested as an alternative. Recently, one study has assessed the prognostic value of the PD-L1 RNA expression in patients with NMIBC (with T1 tumours); however, a heterogeneous population in terms of adjuvant treatment regimen (BCG and mitomycin C) was analysed. The results indicated that high PD-L1 mRNA expression was associated with significantly improved RFS, PFS and cancer-specific survival [11]. Among the included studies, only Aydin et al. [9] provided data for RFS and PFS, and positive PD-L1 expression was not correlated with any survival parameter. This raises doubts as to whether increased PD-L1 expression reflects poor overall prognosis in patients with NMIBC treated with BCG.

Even though PD-L1 does not appear to have utility as a prognostic biomarker for BCG response and overall disease outcomes in patients with NMIB, it might still emerge as a predictive biomarker for checkpoint inhibitors response (PD-L1/programmed cell death protein 1 [PD-1] inhibitors), as this therapeutic approach represent a considerable opportunity in high-risk, BCG unresponsive NMIBC. To date, these conclusions have been based only on publications including patients with MIBC. Increased PD-L1 expression was reported to be predominantly associated with improved objective responses to PD-L1/PD-1 inhibitors [11]. As it was reported in some of the presented studies, an increase in PD-L1 expression after BCG treatment may occur [6]. Theoretically, such patients could significantly benefit from treatment with checkpoint inhibitors. On the other hand, the decrease in post-treatment PD-L1 expression was also observed and, in such patients, anti-PD-L1/PD-1 therapy following BCG failure might be limited [8]. Notwithstanding, it should be emphasised that patients with negative PD-L1 expression could also benefit from checkpoint immunotherapy [12]. Therefore, further clinical trials should be conducted to precisely elucidate the utility of PD-L1 expression as a predictor of checkpoint inhibitors response in patients with failure of BCG therapy.

## Conclusions

The value of PD-L1 expression in predicting BCG response is questionable. Current evidence, based only on retrospective analyses, is inconsistent. The comparability of the results is diminished by the methodological limitations of IHC assessment. Further multicentre, randomised trials are needed to make definitive conclusions.
